# Safety of non-anesthesiologist positive pressure ventilation and sedation/analgesia during cardiac electrophysiology (EP) procedures in high-risk patients with known or risk factors for obstructive sleep apnea (OSA)

**DOI:** 10.1007/s10840-025-02044-5

**Published:** 2025-05-30

**Authors:** John D. Fisher, Thomas Aldrich, Linda Lewallen, Jason Adkins, Mohammad H. Mustehsan, Yvette Ash, Marjan Rahmanian, Suzanne Knowlton, Vanessa Taylor, Marianne O’Shea, Vilma Joseph

**Affiliations:** 1 Montefiore Medical Center, Albert Einstein College of Medicine, 111 E. 210 Th Street, Bronx, NY 10467 USA; 2 Department of Medicine, Montefiore Medical Center - Albert Einstein College of Medicine, Bronx, NY USA; 3 Cardiology (Arrhythmia Or Nursing), Montefiore Medical Center - Albert Einstein College of Medicine, Bronx, NY USA; 4 Pulmonary-Critical Care Divisions, Montefiore Medical Center - Albert Einstein College of Medicine, Bronx, NY USA; 5 Anesthesiology Department, Montefiore Medical Center - Albert Einstein College of Medicine, Bronx, NY USA; 6 Montefiore-Einstein Institutional Procedural Sedation Committee, Montefiore Medical Center - Albert Einstein College of Medicine, Bronx, NY USA

**Keywords:** CPAP, BiPAP, NIPPV, Cath lab, EP lab, Sedation, Procedural, Obstructive sleep apnea, OSA, Non-anesthesiologist

## Abstract

**Abstract:**

Concerns exist about the safety of non-anesthesiologist positive pressure ventilation with sedation/analgesia during cardiac electrophysiology (EP) procedures in high-risk patients with known or risk factors such as obstructive sleep apnea (OSA). This is magnified if the procedures are done outside of intensive care areas or outside of hospital policies and procedures rules.

**Background:**

Noninvasive positive pressure ventilation mask ventilation (NIPPV including continuous or bilevel positive airway pressure—CPAP/BiPAP) with sedation/analgesia is typically limited to hospital units staffed by pulmonary-intensive care or anesthesiology personnel, with monitoring by respiratory therapists or specifically trained nursing staff. NIPPV with sedation has raised concerns if delivered by laboratory staff in procedure rooms, especially in high-risk patients. Literature is sparse on this topic. NIPPV as described is routine at some institutions and prohibited at others. We aimed (1) to test the safety and efficacy of NIPPV with sedation prescribed by cardiologists and administered by trained nurses in a prospective cohort of high-risk patients and (2) to provide data that, if favorable, could lead to revisions of institutional policies.

**Methods:**

We enrolled 50 consecutive consenting patients with known or at high risk for OSA. Three were then excluded (did not qualify, or procedure canceled). Procedures in 47 patients included 21 ICD implants (12 with defibrillation testing), 8 pacemaker implants, 11 ablations, and 7 cardioversions; some patients had combined procedures, e.g., “ablate & pace.” Standard NIPPV settings were used. Staff were trained in general NIPPV device monitoring and management. Data collected included vital signs, O_2_ saturations, hypercapnia, demographics, toleration of NIPPV, and complications.

**Results:**

There were no NIPPV-related complications and no long-term adverse sequelae in the 47 patients who participated in the protocol. No patient required intubation or urgent rescue from an anesthesiologist. Most patients (45) tolerated NIPPV including patients without prior experience.

**Conclusions:**

NIPPV with sedation can be safely delivered in high-risk OSA patients by trained non-anesthesiologist/pulmonary/intensive care personnel in an EP lab setting. Policy and procedure manuals may benefit from revision.

## Introduction

Previous studies have demonstrated the safety of moderate sedation/analgesia delivered by non-anesthesia personnel outside of intensive care units, such as in cardiology laboratories, gastroenterology (GI) procedure rooms, and emergency departments [1–6]. At present, such use of sedation/analgesia is endorsed by many professional societies, including cardiology [1, 4], gastroenterology [5], emergency medicine [6], and anesthesiology [7, 8].  Other important issues remain.

### High-risk Patients  

An additional consideration is the need for respiratory support in patients with known, suspected, or risk factors for obstructive sleep apnea (OSA). These patients are considered high-risk [9] and may require noninvasive positive pressure ventilation (NIPPV) for several reasons including hypoxia, hypercapnia, and arrhythmias [10–14]. A recent retrospective study has supported the use of NIPPV in routine (*not* particularly high-risk) procedures in gastroenterology, interventional radiology, and unspecified cardiac procedures, where duration and depth of sedation tend to be less than for electrophysiologic (EP) studies [15]. We now present a prospective non-randomized study of high-risk (OSA) patients undergoing cardiac electrophysiologic studies delivered by non-anesthesiologists in the EP laboratory.

###   Updating of Policies and Procedures

NIPPV in any patient delivered by non-anesthesiology personnel during EP procedures may conflict with institutional policies and procedures manuals [16, 17]. Relevant guidelines and regulations, e.g., from The Joint Commission (TJC), Centers for Medicare and Medicaid Services (CMS), and New York State, speak to training and equipment for sedation and NIPPV, but not to specific areas or units within the health care facility [18–22].

Our hypothesis is to determine whether NIPPV and sedation administered by cardiologists in patients with OSA will be safe and effective without the need for rescue by anesthesiologists. Our *primary objective* is to demonstrate such safety. The protocol also assesses the ability of cardiologists to select patients who can safely undergo EP procedures.

*Secondary objectives* are to support and improve care by making it possible to perform indicated EP studies that would otherwise be prohibited because of the perceived risk of sedation-associated respiratory complications in the absence of an anesthesiologist.

*An additional secondary aim* is to encourage comparison and possible revision of local and national policies with real-world practices in the electrophysiology (EP) laboratory.

## Methods

**Fig. 1:**
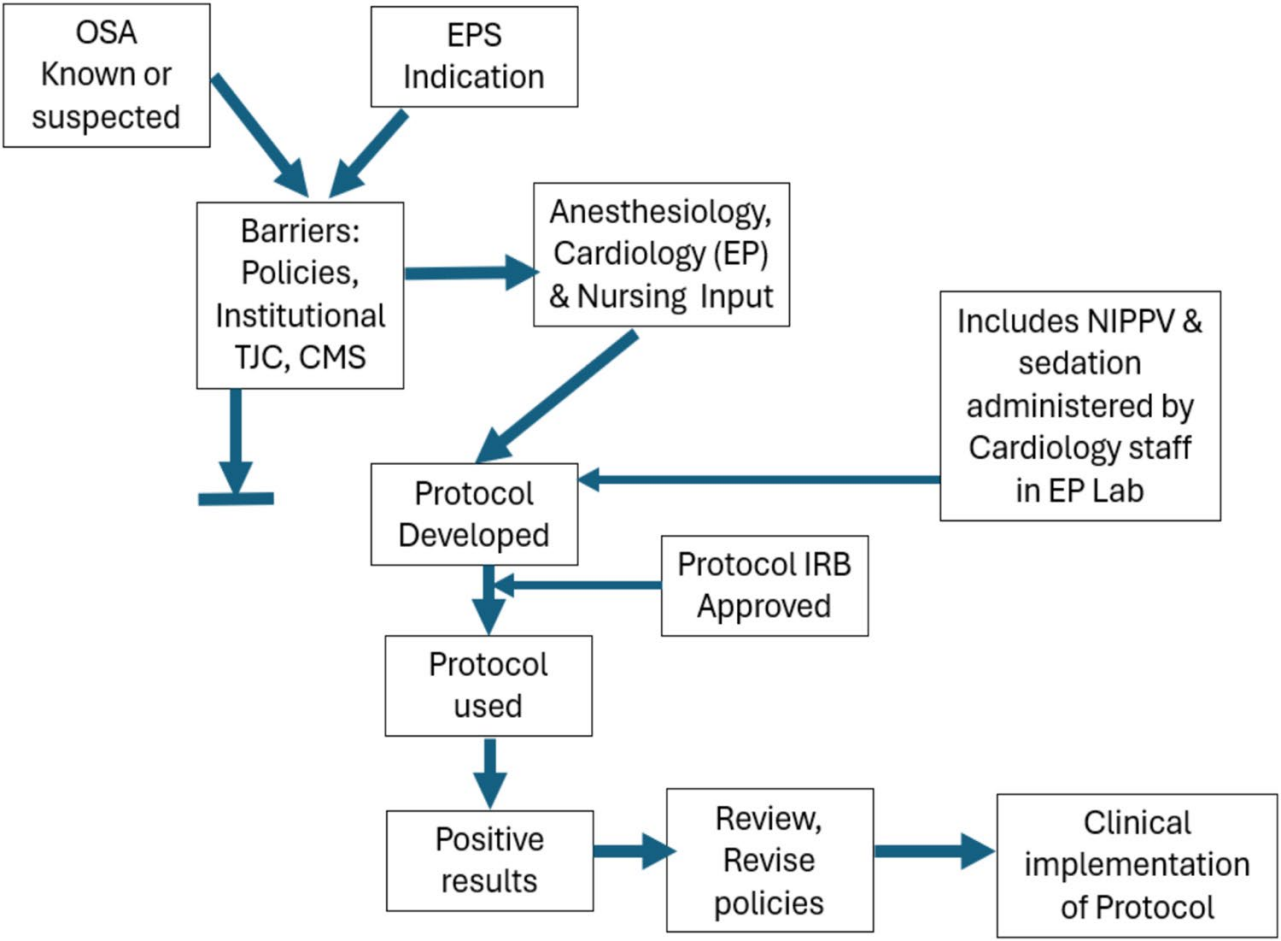
Central illustration. This flow chart depicts the themes and progression of this protocol for the use of NIPPV with sedation administered by cardiology staff in the electrophysiology laboratory/procedure room. Barriers, particularly (obsolete) policies needed to be overcome. The IRB-approved protocol deferred barrier policies, and the positive outcomes led to a review and likely revision of related institutional policies

The protocol was approved by the Montefiore-Einstein Institutional Review Board for the Protection of Human Subjects (IRB), and all patients were fully informed and signed the IRB-approved consent form. We proposed that a pilot study be done with 20 patients to evaluate the feasibility of NIPPV during their EPS procedure. After the initial 20 patients, the IRB recommended increasing the study to 50 patients, without any protocol changes. The process is summarized in the central illustration (Fig. 1).

### Personnel and process

Training of the nurses was confidence-based and was provided by the Department of Respiratory Therapy in conjunction with the Anesthesia Department to assure that the electrophysiology nursing staff were competent and comfortable with the use of the NIPPV machines. During training, each nurse was proctored by a respiratory therapist, and patients were not included in the present study if the nurse was still in the proctoring process. All nurses and physicians passed a certification process including ACLS and education in deep sedation, which included a video module developed and updated by one of the authors (VJ, a senior anesthesiologist). Many nurses had prior CCU/ICU experience, but this was not a pre-requisite.

The training included how to properly apply the mask on patients, titrate the FiO2/oxygen flows, and adjust the mask as needed based on the patient’s needs via simulation and didactic training. Physicians were instructed on the physiology and function of the NIPPV and titration of the parameters and sedation via didactic training by the respiratory therapists. Rather than pre-set flows or doses, continual titration aimed achieving and maintaining target patient responses. Fire prevention and responses [23] were stressed. These, together with respiratory therapy and anesthesia teaching processes, as well as the study protocol formed the syllabus for training.

### Inclusion and exclusion criteria

#### Inclusion criteria

Male or females aged 18 years or older with ≥ 1 of (1) diagnosis of OSA by sleep study; (2) history of observed apnea by bed partner; (3) neck circumference > 18 inches for males or > 16.5 inches for females; (4) patients with BMI > 27.

#### Exclusion criteria

(1) Patients known not to tolerate a CPAP or BiPAP mask; (2) patients not able to give written or verbal consent; (3) patients who did not wish to participate in the NIPPV study. Prior use of nocturnal CPAP or BiPAP at home was not a criterion for inclusion or exclusion. Patients with unstable arrhythmias (the essence of most EP studies) were not automatically excluded.

### Related policies deferred for this protocol by the IRB

Institutional policies were reviewed, with Montefiore-Einstein as an example [16, 17]. These policies call for arterial blood gas (ABG) analysis at 30 min during NIPPV procedures. This is not current practice in most EP, gastroenterology (GI), or other procedures. Policies exclude unstable arrhythmias. However, such arrhythmias are the essence of most EP studies.

*Restraints* are excluded during NIPPV. The positioning straps used in our laboratory procedure rooms were questioned as possibly fitting the CMS or TJC definition of restraints [18–20], and we proceeded to explore this through legal counsel and queries to TJC and CMS. Ultimately, it was determined that TJC and CMS regulations indicate that “positioning” devices used during procedures are distinct from restraints and are permitted [18–20]. See the Discussion section.

Finally, policies excluded sedation plus NIPPV outside of settings such as the various intensive care units. However, EP procedures are performed in cardiology procedure laboratories, not in intensive care units.

### Sequence

#### Recruitment

At their pre-procedure visit, patients who fit the profile of a possible sleep apnea patient were invited to participate in the NIPPV study. Informed and signed consent was obtained in agreeing patients. One objective was to estimate the ability of non-anesthesiologists to select patients who met inclusion criteria and who could safely undergo NIPPV. Therefore, anesthesia consultation was not used routinely prior to the procedure. Anesthesia services are widely available at our institution, but only 2 of 50 participating patients had a pre-procedural anesthesia consultation. All patients underwent institutionally standardized pre-procedure evaluations including ASA and Mallampati-equivalent assessments separately by both nursing staff and cardiology physicians,

#### In the EP laboratory

Patients were brought to the EP lab, and once positioned on the EP table, they were continuously monitored by an ACLS-certified nurse experienced in the use of medications given for moderate/deep procedural sedation. All current EP laboratory registered nursing staff are experienced in catheterization/EP laboratory critical care, as per above.

The patients were assisted with the placement of a NIPPV mask. Oxygen was administered at a sufficient flow rate to bring oxygen saturation (SpO_2_) to 95–98% while the patient was awake; this was designated as the baseline SpO_2_. Arterial lines and blood gases were not used. After an initial period of 5–10 min of spontaneous breathing on oxygen, BiPAP was applied at a setting of 10 cm H_2_O IPAP and 5 cm H_2_O EPAP achieved gradually over 5 min. If nasal airflow did not achieve near-baseline levels, further increases in IPAP and EPAP were instituted in 2 cm H_2_O increments every 5–10 min.

Baseline measurements of heart rate, respiratory rate, chest wall motion (CWM), SpO_2_, and respiratory airflow (Vi) were recorded for 30 s. Then, IV sedation/analgesia was administered and titrated as per our usual protocol [3] to attain a level of sedation that would allow the patient to remain comfortable on the EP table for the duration of the procedure. The sedation target was usually “moderate” but could vary from light/minimal to brief periods of deep sedation, e.g., during implantable defibrillator shock testing.

Medications given for sedation/analgesia in our EP laboratory include midazolam, fentanyl, morphine, and diphenhydramine. Agents, such as propofol and ketamine, cannot be administered by nurses in non-intubated patients in New York State (21). Reversal agents, naloxone and flumazenil, were immediately available.

Non-invasive blood pressure measurements were taken every 5 min; pulse oximetry (SpO_2_), heart rate, and end-tidal carbon dioxide (PETCO2/ETCO) were monitored continuously, with appropriate alarms, and chest wall movement (CWM) was monitored by observation and/or auscultation. Each EP laboratory is equipped with a defibrillator and a bag valve mask if there was a need for increased ventilation. High-flow nasal prongs were available. A kit for intubation was readily available. Respiratory therapy and anesthesiology backup were available.

The physiologic data was displayed in 30-s epochs, and obstructive apneas were identified by comparison of chest wall motion and air/O_2_ flow (Vi) and quantified as to frequency and duration. Episodes of respiratory distress or desaturation (< 88% SpO_2_) were identified and quantified for each subject.

If at any time during the EP procedure, the patient’s SpO_2_ approached 80% for longer than 1 min despite appropriate routine interventions (listed in the next sentence), the EP procedure was suspended, and the NIPPV mask was removed. Such patients were mask-ventilated with the continuation of verbal stimulation, cessation of additional sedation, head tilt, or jaw lift maneuvers until the SpO_2_ was greater than 92%. Procedure failure occurred if the SPO2 could not be raised to ≥ 92%. If the oxygen saturation could be raised above 92% and stable, then at the discretion of the cardiologist, the EP procedure was either terminated (protocol failure) or continued with or without NIPPV in place until the end of the EP procedure (always without if SPO2 fell to ≤ 80% for ≥ 1 min). Similarly, waveform capnography/PETCO2 was followed, and continuation of the procedure was assessed if the waveform changed or PETCO2 dropped to 15–20 mmHg, depending on baseline and rapidity of change and whether up-titration of ventilation restored PETCO2 to ≥ 35 mmHg.

*Recovery* followed current guidelines, which include monitoring the patient in an appropriately staffed and equipped area until they were at or near their baseline level of consciousness and were no longer at sedation-related increased risk for cardiorespiratory depression. Oxygenation, ECG, and blood pressure were monitored.

*The risks* involved with this study were expected to be the same as risks associated with giving moderate/deep procedural sedation and the same as risks that are involved with a standard EP procedure in OSA patients. These risks include, but are not limited to, nausea, vomiting, aspiration, apnea, hypoxia, hypercapnia, possible need for endotracheal intubation, and arrhythmias that could require defibrillation. If any of the above-mentioned adverse events occurred, clinical discretion was used to determine whether the EP study would or would not be completed. It should be noted that induction of arrhythmias is often part of an EP study. Therefore, intentional induction of arrhythmias was not considered as a complication.

Data collected for the purposes of this study but not as selection-inclusion criteria included the following:

1. Patient demographics, such as gender and age, and clinical data, including BMI, neck circumference, type of procedure, length of procedure, and American Society of Anesthesiology (ASA) class (I–VI). In view of known or suspected OSA for the patients in this study, all were assigned a minimum of ASA class II.
  Airway assessment: In lieu of the widely used Mallampati score (class 1–4), Montefiore developed an institution-wide pre-anesthesia sedation system. This involves separate forms for physicians (EP cardiologists for this report) and nurses. Points are often redundant for quality and confirmation purposes; both include anesthesia-sedation history and airway assessment, together with the ability to lie flat, dental condition, mouth opening, neck extendability, obesity, and OSA history. A Mallampati equivalent score (MES) was used in the present report, with 0–2 classes added for each abnormality depending on severity. For consistency, all MESs were assigned by one investigator (JDF) and subsequently reviewed by at least 1 other investigator during the manuscript preparation process.
2. Physiologic data included intra- or post-procedural episodes of hypoxia or respiratory distress, O2 saturation ranges for each patient (baseline and with NIPPV), PETCO2, need for reversal agents, and time to restoration of O2 saturation ≥ 92% after a desaturation event.
3. Clinical data: procedure time, completion or cancellation of procedure, and sedation medications.
4. Patient toleration was defined as completing the procedure with NIPPV.

### Confidentiality

All information collected for this study was considered private in accordance with HIPPA guidelines. While the results of the research study may be published in a scientific journal, all patient identifiers were removed. Patients agreed to this use of data as part of the informed consent process. Protocol, IRB approval, and individual data for each patient are available.

*The main outcome measures* were safety (the number of NIPPV-associated complications) and efficacy (the number of procedures completed vs. those that had to be canceled or interrupted because of respiratory difficulties).

Regarding safety, for this study, we expected no NIPPV-associated complications causing an adverse outcome.

Regarding efficacy, the purpose of the study is to improve the ability to perform EP studies in sleep apnea patients unassisted by anesthesiology or respiratory therapy, but with rapid backup available. Patients who did not sufficiently maintain their oxygenation status, or regain a SPO2 > 92 despite using NIPPV while receiving sedation/analgesia, were considered a failure for the purposes of this study. The IRB predefined a failure rate of 25% to be acceptable.

### Statistical analysis

As indicated in the Methods section, this was a study of 50 consecutive consenting patients with known or at high risk for OSA who agreed to receive sedation for their EPS under the direction of an electrophysiologist. No attempt was made to select for type or number of procedures, gender, procedure time, or other factors. Statistics and analysis were limited to (1) safety: whether the procedure was safely completed from a NIPPV perspective [11, 13–15]; (2) efficacy: actual use of NIPPV and delivering desired levels of sedation; (3) descriptive matters such as patient and procedure details; and (4) a review of local and national guidance documents.

## Results

The results are detailed in Tables 1, 2, 3, 4, and 5. Fifty patients were enrolled in this study (Table 1). An anesthesiology attending (VJ) retrospectively reviewed the patient selection post-procedurally at the conclusion of the whole study, as part of manuscript preparation. Three patients were excluded before the procedure: two were found not to qualify, and for one, the procedure was canceled (spontaneous reversion from atrial fibrillation to normal sinus rhythm). Of the remaining 47 patients, two did not tolerate the mask; thus, 45 (90%) completed the protocol. The two who did not tolerate NIPPV both had morbid obesity and were overtly anxious. One was a man aged 45, with no previous mask experience and a BMI higher than the study average at 45. He was anxious and required more initial sedation, and the actual procedure had not begun when the patient asked to remove the mask. The other patient was a woman 65, no previous mask experience, also with a high BMI of 47, and also required more initial sedation. Neither had abnormal changes in monitored data. Both had a history of hypertension and diabetes, common in the study population. Neither had known coronary artery disease.

**Table 1:** Patient characteristics

	Number (%)	Mean +/− SD	Comment
Patients consented	50 (100)		
Included	47 (94)		
Excluded	3 (6)		Did not qualify (2) or procedure not done
ASA Class		2.8 +/−.048	
Mallampati*		2.7 +/−.045	
Tolerated mask and NIPPV	45 (90)		90%
Women	19 (38)		
Age (years)		61.9 +/− 10.7	
Men	28 (56)		
Age (years)		62.4 +/− 10.7	43
BMI		38.5 +/− 7.9	
Number ever smoked: yes	18 (36)		5 Unknown
Prior mask use	8 (16)		
LVEF		46.2 +/− 15.2	
Heart failure	25 (50)		
Hypertension	35 (70)		
Diabetes	22 (44)		
Coronary disease	13 (26)		
Pulmonary-COPD	6 (12)		
Implanted defibrillator. (prior)	11 (22)		
3 or more comorbidities	30 (60)		

*SD*, standard deviation; *NIPPV*, noninvasive positive pressure ventilation; *BMI*, body mass index; +/0, yes/no; *LVEF*, left ventricular ejection fraction; *COPD*, chronic obstructive pulmonary disease. Heart failure includes all types. Diabetes includes all types. *Mallampati: we used our Institution’s standard airway assessment, forms that include oropharynx examination, and more (see text)

**Table 2:** Procedures

Procedure	Number (%)
Patients total	47 (100)
Pacemaker	8 (17)
ICD = defibrillator (new)	21 (45)
Defibrillator shock testing (DFT) at implant	12 (25)
Diagnostic EPS	4 (9)
Ablation	11 (23)
Ablation of atrial flutter	5 (11)
Ablation of AVNRT	4 (9)
Ablation of AF	1 (2)
Ablation of AV node	1 (2)
Ablation + pacemaker	1 (2)
DCCV = cardioversion	7 (15)

*EPS*, electrophysiologic study; *AVN*, atrioventricular node; *AVNRT*, AVN reentry tachycardia; *AF*, atrial fibrillation; *AFL*, atrial flutter; defibrillator: *ICD*, implantable cardiac defibrillator; *DCCV*, direct current cardioversion

**Table 3:** Multiple procedures at same day/session (immediately sequential or simultaneous)

Procedures at same session	Number patients (%)
Total number patients	47 (100)
ICD extraction + new ICD	1 (2)
ICD + AFL ablation	2 (4)
ICD + AVNRT ablation	1 (2)
ICD + EPS	2 (4)
TEE + AVNRT ablation	1 (2)
Implantable loop recorder + AVNRT ablation	1 (2)
TEE + AVN ablation + pacemaker	1 (2)
DCCV + pacemaker	2 (4)
DCCV + EPS	1 (2)
DCCV + TEE	5 (11)
Total	17 (36)

Procedure: cardiac procedure during which NIPPV was used. Extraction: removal of ICD system including lead(s). Ablation: procedure to treat arrhythmia by catheter delivery of radiofrequency energy. *TEE*, trans-esophageal echocardiography; *EPS*, electrophysiologic study; *DCCV*, direct current cardioversion. Note: Five patients had TEEs before DCCV; masks were used only after TEE was finished. ICD procedures may include briefly deeper sedation during ventricular arrhythmia induction and defibrillation

**Table 4:** Patient toleration and vital signs during CPAP/NIPPV

	Number (%)	Mean, +/− SD	Comment
Total pts	47 (100)		
Tolerated	45 (96)		
Baseline SpO_2_		98.2 +/− 2.2	
Minimum SpO_2_		95.7 +/− 2.6	
Duration of minimum SpO_2_ (min)		6.9 +/− 12	Driven by 1 patient, 85 min at minimum: 95% baseline was 95%
SpO_2_2 dropped to < 93	5 (11)		Baselines in these: 98, 90, 97, 100, 96. Lowest Sat recorded: 90% in 2 patients
SpO_2_2 < 80	0 (0)		
Blood pressure (BP)			
Baseline BP systolic mmHg	47 (100)	137 +/− 23	1 systolic > 180 5 diastolic > 100
Minimum systolic BP	45	112 +/− 22	*P* < 0.005 vs baseline
Max increase systolic > 20 mmHg	16	167 +/− 28	1 patient > 200; baseline was 196
Max decrease systolic > 20 mmHg	26	109 +/− 23	9 pts < 100 mmHg None needed pressors
PETCO2 baseline	31 (69)	27.3 +/− 7.3	31 of 45 had PETCO2 done
PETCO2 minimum	31 (69)	23.4 +/− 6.9	PETCO2 ever < 20 in 4
Pts with PETCO2 ever < 15	3 (9) Of 31		Treated: ventilation increased as needed
Baseline Aldrete score	45 (100)	9.9 +/− 0.3	
Post-procedure Aldrete score	45 (100)	9.6 +/− 0.4	
Complications related to NIPPV	0		
Reversal agents used	0		
Procedures terminated	0		

*NIPPV*, noninvasive positive pressure ventilation; *PETCO2*, partial pressure end-tidal carbon dioxide (also called ETCO2); *SpO*_*2*_, oximetry peripheral capillary oxygen saturation

**Table 5:** Sedation medications and procedure times

	Morphine (mg) +/− SD	Fentanyl (mcg) +/− SD	Cumulative morphine PO dose equivalent (mg)	Midazolam (mg) +/− SD	Diphenhydramine (mg) +/− SD	Procedure duration (min) +/− SD
No. (Pts) (Total 47)	7*	44	46	36	12	47
Dose mean	8.30 +/− 2.50	158.2 +/− 86.0	41.0 +/− 23.4	7.30 +/− 5.0	41.7 +/− 12.3	186.9 +/− 155.4
Dose/min	0.08 +/− 0.10	1.46 +/− 1.80		0.07 +/− 0.12	0.47 +/− 0.33	

Medication details missing on one patient. Procedure duration missing in one patient. *PO*, oral; *No*, number of patients (Pts)

^*^Five of seven patients also received fentanyl

The most common procedure was a defibrillator (ICD) implant (Table 2).

As anticipated from the entry criteria, the patients were generally obese (mean BMI 38.5); 30 had ≥ 3 co-morbidities including ASA Class ≥ III and airway abnormalities; there was a high prevalence of hypertension, diabetes, and depressed left ventricular ejection fractions (Table 1).

The baseline SpO_2_ after nasal oxygen and before NIPPV was 98%, and the mean minimum SpO_2_ during sedation was 96%. In five patients, the minimum SpO_2_ was less than 93%; the lowest SpO_2_ was 90% in two patients. One of these had a baseline of 90% and maintained this without intervention and was considered an exception. The other patient responded to supportive measures (physical maneuvers). Both completed their procedures. Thus, no patients were failures by protocol SpO_2_ definition.

The mean baseline PETCO2 was 27.3 mmHg (Table 4) in 31 patients. Patients with a diagnosis of OSA, as defined for this protocol, had an insignificantly lower baseline PETCO2 (*p* < 0.37), but only seven patients contributed to this, and larger numbers could make a difference. PETCOs did not differ significantly among baseline and minimal levels for all groups: baseline and minimal for OSA patients, or for the whole group, or between baseline and finals of the OSA and the whole groups. In all these comparisons, PETCO’s means were between 23.7 and 27.3 (*p* < 0.37 to < 0.95 +/− 6.0–7.3). In three patients, PETCO2 dropped briefly below 15 mmHg, and in each case, the PETCO2 was promptly restored to baseline or higher by increased ventilation.

No procedure needed to be discontinued. The most common sedation medications were fentanyl and midazolam (Table 5). No reversal agents were used. There were no arterial blood gasses drawn or sent.

The 50 cases were distributed among six different proceduralists, only one of whom (JDF) was an author of this manuscript.

The mean procedure duration was 187 min, ranging from 19 min for a non-invasive programmed stimulation (NIPS) to 660 min for a complex EP study and ablation. Brief deep sedation during shocks for cardioversion or defibrillator testing in 33 patients were all done without adverse effects. There were no complications.

## Discussion

*Our main finding* in this prospective study was that intravenous sedation/analgesia and NIPPV delivered by trained EP lab nurses and procedural electrophysiologists appears to be safe in a high-risk population of patients with known or suspected OSA. These results complement a recent large series [15] with a similar though retrospective protocol with fewer details, in OSA patients having GI, interventional radiology, or unspecified cardiac procedures. In that study, including cardiac catheterization, procedures tend to be briefer, arrhythmias were avoided if possible, and sedation levels were often minimal to moderate rather than moderate with occasional periods of deep sedation.

### Historical context

During the early decades of EP testing, it was usual to do the procedures with patients in the “post-absorptive, non-sedated state.” As cases became longer and more complex, or required delivery of shocks, sedation delivered by EP lab personnel became the norm [1]. During the 1990 s, there were concerns about safety and scope of practice. These concerns were addressed by guidelines from the Heart Rhythm Society [1] (at that time called NASPE) and the American Society of Anesthesiologists, which published guidelines for sedation administered by non-anesthesiologists [1, 7, 8].

### Policies, procedures, and guidelines

Patients with conditions such as obesity and OSA are prone to develop arrhythmias that may be indications for EP studies [9–13]. A combination of NIPPV and sedation/analgesia in the EP laboratory, delivered by EP staff who are trained in NIPPV, has become routine in some laboratories, but hesitancies remain. Data from a retrospective study [15] and the present prospective study appear to demonstrate a high degree of safety and efficacy with cardiac laboratory staff delivery of NIPPV and sedation. This held true in the present study in high-risk patients with OSA, including those with multiple co-morbidities.

Our institutional guidelines [16, 17] call for routine arterial blood gas (ABG) analysis at 30 min during NIPPV procedures. These requirements arose from appropriate practices in intensive care units. This is not current practice in most EP, GI, or other procedures. For this protocol, ABGs were to be done at the discretion of the proceduralists, and during this study, none were drawn.

#### Restraints

The wording of TJC and CMS regarding restraints is similar [17–19]: “A restraint does not include devices…or methods that involve the physical holding of a patient for the purposes of conducting routine physical examinations or tests.” In the EP lab, positioning straps/holders are used to maintain position, limit mobility, or temporarily immobilize a patient during medical, diagnostic, or surgical procedures. Thus, the hospital policy forbidding restraints does not apply. This clarifies a subtle but critical difference. Positioning devices in our laboratory are partly as reminders to the patient that positional stability is critical to accurate arrhythmia mapping and ablation and to limit major movements during delivery of shocks.

### Current status

Anesthesiology presence during an EP study is always welcome. Scheduled procedures are done according to risk assessment and anesthesia schedule. However, some urgent procedures in high-risk patients cannot wait for a routine opening in the anesthesia schedule [4, 6]. Anesthesia availability at our institution has increased significantly in recent years, but 100% coverage is not yet possible except on an emergent basis. As an added consideration, we found that over a decade, $5 million dollars was saved by performing cardiac procedures with sedation but without (routinely) an anesthesiologist [2].

### Implications

The results of the present study were gratifying. Given the lack of complications or any hypoxia below SpO_2_ 90%, it may be reasonable to consider trained EP nurse-administered NIPPV in many or most EP cases with OSA where anesthesiology personnel is not present.

Continual practice is needed for skills maintenance. It would be desirable to develop training criteria including simulation for prescribing sedation plus NIPPV in procedural laboratories by cardiologists and administration by registered nurses. This has been successful at our institution, led by anesthesiologist author VJ. Based on our experience, such training need not be exhaustive if staff are already competent in intravenous sedation/analgesia.

### Limitations

We note the absence of complications, but we do not have a randomized control group to determine if outcomes would be different without NIPPV or with anesthesia services for all. Larger retrospective studies [15] in lower-risk patients have indicated that urgent-emergent calls for anesthesia rescue are rare but can occur at 1–2% of cases. Thus, there is a need for a careful selection of patients suitable for cardiologist-prescribed and registered nurse-administered sedation, especially with NIPPV. Our protocol called for suspension of the EP protocol for SpO_2_ < 80 for ≥ 1 min despite routine interventions: different numbers might have been chosen, but none of our patients came near the protocol limits.

#### Extrapolation

We have demonstrated the safety of EP procedures using NIPPV in high-risk OSA patients, but this study did not include other high-risk patients or lower-risk patients. Such patients often undergo procedures with sedation delivered by non-anesthesia personnel, without NIPPV, in accordance with current recommendations [e.g., 1,4,7,8]. Our study focused on high-risk patients with OSA. The results may not apply to patients with active heart failure, or other active instabilities, and for these, we have been quick to engage anesthesia services. We do not have data on the use of NIPPV in OSA patients during GI, interventional radiology procedures, or other.

### Conclusions

1. NIPPV can be safely administered by trained non-anesthesiologists in high-risk OSA patients who are undergoing sedation for electrophysiology procedures.
2. The results of the present study support the development of a randomized or matched cohort trial comparing electrophysiologist-directed NIPPV and sedation with anesthesiologist-monitored anesthesia care (MAC).
3. It may be appropriate to review institutional policies related to NIPPV in procedure laboratories. This applies to costs, safety, and medico-legal implications.

## Definitions

**Moderate sedation****/analgesia**: A drug-induced depression of consciousness, during which patients respond purposefully to verbal commands and/or physical stimulation. No interventions are required to maintain a patent airway, and spontaneous ventilation is adequate
**Deep sedation/analgesia**: A drug-induced depression of consciousness, during which patients cannot be easily aroused but respond purposefully to repeated or painful stimulation. The ability to independently maintain ventilatory function may be impaired. Patients may require assistance in maintaining a patent airway, and spontaneous ventilation may be inadequate. Cardiovascular function is usually maintained
**NIPPV**: Noninvasive positive pressure ventilation refers to the delivery of positive pressure ventilation through noninvasive interface (e.g., nasal mask, face mask, or nasal prongs rather than an invasive interface (endotracheal tube, tracheostomy). Major forms of NIPPV include CPAP and BiPAP
**CPAP**: Continuous positive airway pressure ventilation
**BiPAP**: Bi-level positive airway pressure (different pressures for inspiration and expiration)
**IPAP**: Inspiratory positive pressure
**EPAP**: Expiratory positive pressure
**OSA, OSAS**: Obstructive sleep apnea syndrome
**EPS**: Electrophysiologic studies
**EP**: Electrophysiology or electrophysiologic
**BMI**: Body mass index
**PETCO2**: Partial pressure of end-tidal carbon dioxide (same as ETCO2)
**ASA**: American Society of Anesthesiologists
**TJC**: The Joint Commission
**CMS**: Centers for Medicare and Medicaid Services
